# The growth of *Chlamydomonas reinhardtii* as influenced by high CO_2_ and low O_2_ in flue gas from a silicomanganese smelter

**DOI:** 10.1007/s10811-014-0357-8

**Published:** 2014-06-15

**Authors:** Leiv M. Mortensen, Hans R. Gislerød

**Affiliations:** Department of Plant and Environmental Science, The University of Life Sciences, 1432 Ås, Norway

**Keywords:** Biomass, Microalgae, Nitrogen oxides, Oxygen concentration (O_2_)

## Abstract

The aim of this study was to find an inexpensive and environmentally friendly CO_2_ source for growing the hydrogen-producing microalgae *Chlamydomonas reinhardtii*. The effect of different flue gas concentrations from a silicomanganese smelter on the growth of these algae at a photon flux density of 200 μmol photons m^−2^ s^−1^ applied 24 h day^−1^ was studied. First, the algae were grown in a laboratory at 1.2, 6.8 and 17.1 % (*v*/*v*) pure CO_2_ gas mixed with fresh air. After 5 days of growth, the dry biomass per litre algal culture was slightly higher (17 %) at 6.8 % CO_2_ as compared to at 1.2 % CO_2_. A further increase to 17.1 % CO_2_ decreased the biomass by about 40 %. Then, the flue gas from a silicomanganese smelter was used as a CO_2_ source for growing the algae. The flue gas was characterized by a high CO_2_ concentration (about 17 % *v*/*v*), low oxygen concentration (about 4 %), about 100 ppm NO_*x*_ and 1 ppm SO_2_. The culture medium bubbled with undiluted flue gas contained about 490 mg L^−1^ dissolved CO_2_ and 4.0 mg L^−1^ dissolved O_2_, while the lowest flue gas concentration contained about 280 mg L^−1^ CO_2_ and 7.1 mg L^−1^ O_2_. Undiluted flue gas (17.4 % CO_2_) decreased the biomass of the algae by about 40 % as compared with 4.8 % pure CO_2_ gas or flue gas diluted to a concentration of 6.3 % CO_2_. Flue gas diluted to give 10.0 % CO_2_ gave less reduction in the growth of the algae (22 %). It was concluded that the high CO_2_ concentration itself caused the growth reduction and not the air pollutants, and the very low O_2_ concentrations in the growth medium could not counteract this negative effect.

## Introduction

Hydrogen is recognised as a promising future energy alternative because it produces no carbon dioxide that contributes to the greenhouse effect when combusted (IPCC 2013). Today, conventional hydrogen production is energy-intensive, and more environmentally friendly production by means of biological processes is therefore of great interest (Jo et al. 2006; Skjånes et al. 2007). The single-cell green alga *Chlamydomonas reinhardtii* is known to produce hydrogen when starved of sulfur under anaerobic conditions (Melis et al. 2000; Nguyen et al. 2011; Geier et al. 2012). Hydrogen is released through the action of a hydrogenase enzyme that receives the energy from electrons from the breakdown of starch or splitting of water in photosystem II. Before the anaerobic hydrogen-producing stage, the microalgae are grown under aerobic conditions: sunlight with CO_2_ and nutrients. Today’s atmospheric CO_2_ concentration of around 400 ppm strongly limits the algae growth, and additional CO_2_ should be supplied throughout the production phase (Geier et al. 2012). It has also been found that *C. reinhardtii* grown at high CO_2_ concentrations subsequently produces more hydrogen than when grown at low CO_2_ levels (Geier et al. 2012). In order for the production to be environmentally friendly, waste CO_2_ from industrial flue gas should be used, which also contributes to cleaning the emissions. Several studies have investigated the effect of flue gases on the growth of microalgae (Douskova et al. 2009; Kastánek et al. 2010; Borkenstein et al. 2011; Lara-Gil et al. 2014). Depending on the species and the content of different pollutants in the flue gas, the growth varied greatly compared with using pure CO_2_ gas. Although *C. reinhardtii* has been subjected to extensive investigation, the effect of flue gas seems to have been little studied in this species. Bark (2012) showed that *C. reinhardtii* could be grown in simulated flue gas at a concentration of 15 % CO_2_/100 ppm NO; however, the growth was better at lower CO_2_ concentrations. A CO_2_ concentration of 4.5 % was found to be optimal for the growth when the effect of concentrations between 1 and 6 % was studied in *C. reinhardtii* (Daria Markina, unpublished results). In order to maximise the conversion of solar energy to biomass, it is important to search for an industrial emission that stimulates growth to the same extent as pure CO_2_. Since extensive cleaning devices have been installed during recent years, the emissions from a silicomanganese smelter in Norway are recognised as being very clean (www.eramet.no). Highly efficient combustion processes produce emission gas containing up to 20 % CO_2_ accompanied by O_2_ in concentrations down to 1 %. Such high CO_2_ concentrations are rarely obtained in industrial emissions. The combination of high CO_2_ and low O_2_ concentrations is of particular interest for plant production, since combination of high CO_2_ and low O_2_ decreases photorespiration and increases photosynthesis in C_3_ plants as well as in microalgae (Ramazanov and Cardenas 1992; Kliphuis et al. 2011). In studies of the effect of CO_2_ concentration on the growth of microalgae, CO_2_ gas is usually mixed with air, causing dilution of the O_2_ content. If 20 % CO_2_ is added to the air, the resulting O_2_ concentration would be around 17 %, while the emission from the silicomanganese smelter in our study could reach 20 % CO_2_ combined with 1 % O_2_. Our study therefore focused on the effect of the CO_2_ concentration and air pollutants in the flue gas as well as on the O_2_ concentration. The study was a combination of laboratory experiments with pure CO_2_ gas mixed with fresh air and experiments with flue gas at the site of the silicomanganese smelter. Since previous results have shown that concentrations above 1 % CO_2_ increase the growth of this alga, around 1 % CO_2_ was chosen to be the lowest concentration in this study (Markina, unpublished results).

## Materials and methods

*Chlamydomonas reinhardtii* strain SAG 34.89 from Georg-August-Universität Göttingen (Germany) obtained from the NIVA culture collection, Norway, was used in the experiments. The algae were stored on Petri dishes covered with TAP medium 1.5 % agar (Gorman and Levine 1965). The algae were grown in tap water-based high-salt Sueoka medium (Sueoka 1960). Sodium bicarbonate was used at 10 mM in order to buffer the culture medium since pH otherwise would become very low due to ammonium uptake by *C. reinhardtii*. The addition of bicarbonate typically increased pH of the culture medium by 0.6 units. The microalgae were grown in 1.0-L clear plastic bottles (80 mm inner and 82 mm outer diameter) filled with 0.85 L of growing medium (filled to 17 cm). Twelve bottles were placed in a row adjacent to each other. The light was supplied from one side by four cool white fluorescent tubes (Osram L36W/840) placed about 10 cm in front of the bottles. A photon flux density (PFD) of 200 ± 10 μmol photons m^−2^ s^−1^ was given 24 h day^−1^ and was measured by a LI-COR Model LI-250 instrument with a quantum sensor (400–700 nm). The CO_2_ concentration was measured using a Vaisala CO_2_ transmitter (type GMT221, range 0–5 %) or a Vaisala GMP instrument with a sensor in the range 0–20 %. The CO_2_ concentration was recorded once in an hour. The temperature was measured by copper-constantan thermocouples and was recorded hourly using a Campbell AM25T multiplexer.

The different CO_2_ concentrations with pure CO_2_ gas were established by mixing food grade CO_2_ with fresh air. The CO_2_ gas flow was determined by capillaries with defined resistances. The gas pressure was defined by the height of a water column. In this way, a very accurate CO_2_ flow could be added to a constant rate of fresh air produced by air pumps (Resun ACO-001, ACO-004). The different gas mixtures were bubbled through 0.3-cm-inner diameter plastic tubes to the bottom of the bottles at a rate of approximately 100 L h^−1^. All treatments in all experiments included three parallel bottles with 0.85 L culture. The initial algae concentration was 0.2 g dry weight L^−1^ medium in the different experiments. For practical reasons, the start culture had been stored in darkness at 5 °C; however, this seemed not to reduce the activity of the microalgae since no lag period was observed at start of the experiments.

### Laboratory experiments

*C. reinhardtii* was grown in pure CO_2_ gas mixed with fresh air to give three CO_2_ concentrations (1.2 ± 0.4, 6.8 ± 1.0 and 17.1 ± 2.4 %). The experiment lasted 6 days and the temperature was 23.8 ± 1.5 °C. In order to see if an acclimation to high CO_2_ concentrations (6.5 ± 0.3 and 16.2 ± 0.7 %) took place, the algae were grown in another experiment for 6 days before dilution of the cultures and continuation of the experiment for another 5 days. In this experiment, the turbidity (FTU) was measured by a Hanna instrument (HI 93703). The measurements were carried out in the range 0–50 FTU (the linear phase) by diluting the algal culture if necessary. The temperature in this experiment was 22.0 ± 1.7 °C. The variation in the temperatures was due to no cooling devices in the algal growing room.

#### Experiment with flue gas from a silicomanganese smelter

An experimental set-up was established in a silicomanganese smelter (Eramet Norway Kvinesdal; www.eramet.no). The melting furnaces are run on electricity, and the flue gas from the furnaces is cleaned in order remove polycyclic aromatic hydrocarbons (PAHs) and mercury before emission as cleaned flue gases through the chimney. In 2012, the emissions from the furnaces amounted to 208,000 t of CO_2_, 88.5 t of NO_*x*_ (95 % NO), 4.12 t of SO_2_ and 2.68 kg of Hg (www.norskeutslipp.no; Eramet Norway Kvinesdal AS). Flue gas from the chimney was sucked by pumps (Resun ACO-008A) with a capacity of 6.9 m^3^ h^−1^ through two 100-L plastic tubs in series for condensation of the water vapour. Then, the flue gas was diluted by mixing with fresh air in order to establish two CO_2_ concentrations in addition to the CO_2_ concentration in the undiluted flue gas. The mean concentrations throughout the 4-day experimental period were 6.3 ± 1.0, 10.0 ± 1.6 and 17.4 ± 2.9 % in the three flue gas treatments. This corresponded to mean O_2_ concentrations of around 15, 11 and 4 %, respectively. The corresponding NO_*x*_ concentrations were 36 ± 6, 58 ± 10 and 102 ± 13 ppm, the SO_2_ concentrations were 0.4 ± 0.0, 0.6 ± 0.1 and 1.1 ± 0.1 ppm, while the H_2_S concentrations were 0.3 ± 0.0, 0.5 ± 0.1 and 0.8 ± 0.1 ppm, at the different flue gas concentrations, respectively. The measured CO concentration was <5 ppm in the undiluted flue gas. The pollutants were measured using Dräger gas detection tubes (Accuro, Dräger Safety, Germany). The concentration of Hg was not measured during the experiment, but it has been reported to vary between 1 and 5 μg m^−3^ and can be calculated from the yearly emission data (www.norskeutslipp.no). In addition to the different flue gas treatments, a control treatment was established with pure CO_2_ gas at a concentration of 4.8 ± 0.8 %. The O_2_ concentration in the algae culture was measured using an Odeon OPTOD sensor for dissolved oxygen. Light was provided by fluorescent tubes as described above. The mean temperature during the experimental period was 28.7 ± 0.5 °C, a temperature that was the result of the hot environment in a room adjacent to that of the melting furnaces.

In the laboratory experiment, the dry weight of the cells was measured by filtering 0.3–2.0 ml of algal culture through 0.7-μm-pore size, 25-mm-diameter Whatman GF/F glass microfiber filters to collect the biomass. The filters with the algae were washed with 20 mL distilled water to remove adhering salts and then were dried at 104 °C for 4 h. The dry weight was calculated by subtracting the dry weight of the clean filter from the dry weight of the filter with algae. In the experiment at the smelter site, the dry weight was measured by vacuum filtering 10 or 20 mL of culture through a 90-mm filter (Whatman GF/B) and by drying it in an oven for 4 h at 100 °C. No pore size of this filter was given; however, all algal cells remained on the filter since no colouration of the filtered water was observed. The number of cells and cell diameter were counted using a cell counter (Multisizer 4 Coulter Counter, Beckman Coulter). Since the algae were not grown aseptically, it was useful to study the cell size distribution in order to identify if other microorganisms were present in the cultures.

The data were analysed using the SAS ANOVA procedure (SAS Institute, Inc., USA) based on the bottles as replicates (*n* = 3).

#### Dissolved CO_2_ in the growth medium

**Fig. 1:**
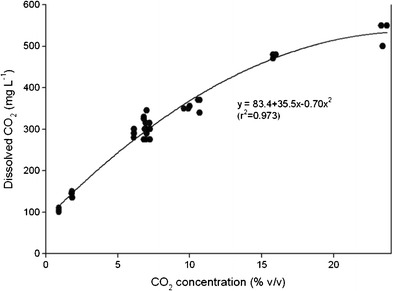
The effect of CO_2_ concentration on dissolved CO_2_ in the growth medium (*n* = 3, SE)

For algae growth, the concentration of dissolved CO_2_ in the nutrient medium is important and not the concentration of CO_2_ in the air bubbled into the culture. Recently, the dissolved CO_2_ in the growth medium was measured with the same algal growing system as used in the present study (Mortensen and Gislerød 2014). Based on these data, a regression analysis was done (Fig. 1). The results showed a progressive increase in the dissolved CO_2_ concentration from about 100 to about 500 mg L^−1^ with increasing CO_2_ concentration from about 1 % up to about 24 %. The regression equation (order 2) was found to be *y* = 83.4 + 35.5*x* − 0.70*x* ^2^ (*r* ^2^ = 0.973). Parallel to this increase, the pH decreased from 7.6 to about 6.5. The measurements were done at 23 °C. The dissolved CO_2_ as measured at 7.0 % CO_2_ was found to be about the same in the temperature range 23–33 °C.

## Results

### Laboratory experiments

**Fig. 2:**
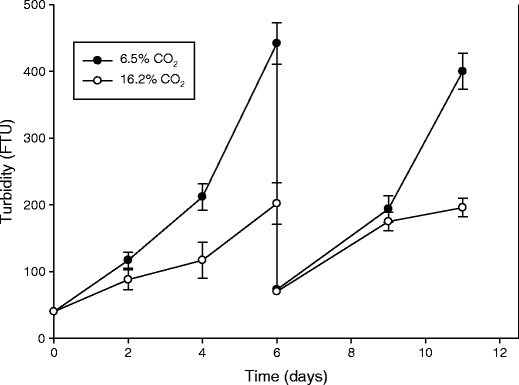
The mean turbidity of *C. reinhardtii* grown at two CO_2_ concentrations at a photon flux density of 200 μmol photons m^−2^ s^−1^ and a temperature of 22.0 °C for 11 days. After 6 days, the cultures were diluted and allowed to grow for another 5 days (*n* = 3, ±SE)

In the first experiment, the dry biomass of the algae (g L^−1^) was increased by 17 % when the CO_2_ concentration was increased from 1.2 to 6.8 %, while a further increase to 17.2 % CO_2_ decreased it by around 40 % (Table 1). The dry weight and diameter per cell were unaffected by the CO_2_ concentration. The pH at end of the experiment decreased from 7.2 to 6.4 when the CO_2_ concentration was increased from the lowest to the highest level. In the second experiment, the turbidity increased in the two treatments (6.5 and 16.2 % CO_2_) over 6 days of growth without any lag phase (Fig. 2). After 6 days, the cultures were diluted and the cultures continued to grow at about the same rate as before dilution. No significant acclimation to high CO_2_ seemed to take place. The growth rate was significantly higher at 6.5 % compared to 16.2 % CO_2_. In this experiment, the dry weight was only measured after 6 days (1.03 ± 0.11 and 0.47 g L^−1^ at 6.5 and 16.2 % CO_2_, respectively).

**Table 1:** The effect of different concentrations of pure CO_2_ on mean the dry biomass, cell diameter and dry weight (*n* = 3, ±SE) of *C. reinhardtii* grown 5 days at a photon flux density of 200 μmol photons m^−2^ s^−1^ and a temperature of 23.8 °C

CO_2_ conc. (%)	pH	Biomass (g L^−1^)	Cell diameter (μm)	Cell w. (рg cell^−1^)
1.2 ± 0.4	7.2 ± 0.1	1.00 ± 0.03	6.6 ± 0.8	1,184 ± 21
6.8 ± 1.0	6.6 ± 0.1	1.17 ± 0.05	7.4 ± 0.1	1,215 ± 35
17.1 ± 2.4	6.4 ± 0.0	0.68 ± 0.15	5.7 ± 0.9	1,258 ± 63
*F* values and significance levels				
	140***	8.52**	2.01^#^	3.58^#^

*F* values and significance levels are given

^#^ *p* > 0.05; *p < 0.05; **p < 0.01; ***p < 0.001

### Flue gas experiment

Undiluted flue gas (17.4 % CO_2_/102 ppm NO_*x*_) decreased the dry biomass (g L^−1^) of the microalgae after 4 days of growth by about 40 % as compared with 4.8 % pure CO_2_ gas or flue gas diluted to a concentration of 6.3 % CO_2_/36 ppm NO_*x*_ (Table 2). Flue gas diluted to give 10.0 % CO_2_/58 ppm NO_*x*_ gave less reduction in the growth of the algae (22 %). The cell diameter was the same in all treatments (Table 2) as well as the dry weight per cell (results not presented). The experiment was repeated with two of the treatments, undiluted flue gas and 4.8 % pure CO_2_ gas. After 4 days, the biomass concentration was 1.11 ± 0.07 g L^−1^ (*n* = 3, ±SE) in the flue gas treatment and 1.87 ± 0.03 g L^−1^ (*n* = 3, ±SE) in pure CO_2_ gas treatment. This was the same decrease in growth (about 40 %, *p* < 0.001) by undiluted flue gas as in the first experiment. Increasing the flue gas concentration from 6.5 to 17.4 % CO_2_ decreased the O_2_ concentration in the culture from 7.4 to 4.0 mg L^−1^.

**Table 2:** Effect of different concentrations of flue gas (FG) and pure CO_2_ gas on mean dry biomass (g L^−1^) and cell diameter of *Chlamydomonas reinhardtii* after 2 and 4 days of growth in a silicomanganese smelter at a photon flux density of 200 μmol photons m^−2^ s^−1^ (*n* = 3, ±SE)

Treatment	NO_*x*_ (ppm)	SO_2_ (ppm)	H_2_S (ppm)	Dissolved CO_2_ (mg L^−1^)^a^	Dissolved O_2_ (mg L^−1^)	pH	Biomass (g L^−1^)		
							2 days	4 days	Cell diam. (μm)
17.4 ± 2.9 % CO_2_ FG	102 ± 13	1.1 ± 0.1	0.8 ± 0.1	489	4.0 ± 0.3	6.3 ± 0.1	0.45 ± 0.04	1.28 ± 0.03	6.6 ± 0.1
10.0 ± 1.6 % CO_2_ FG	58 ± 10	0.6 ± 0.1	0.5 ± 0.1	368	5.9 ± 0.0	6.4 ± 0.0	0.56 ± 0.04	1.67 ± 0.06	6.1 ± 0.2
6.3 ± 1.0 % CO_2_ FG	36 ± 6	0.4 ± 0.0	0.3 ± 0.0	279	7.1 ± 0.5	6.6 ± 0.2	0.63 ± 0.08	1.92 ± 0.05	6.3 ± 0.1
4.8 ± 0.8 % pure CO_2_	0 ± 0	0.0 ± 0.0	0.0 ± 0.0	237	7.4 ± 0.5	6.6 ± 0.1	0.63 ± 0.04	2.14 ± 0.23	6.1 ± 0.2
*F* values and significance levels									
					50.9***	7.12*	7.42*	16.6**	1.37^#^

^a^Values of dissolved CO_2_ was calculated by the formula in Fig. 2

*F* values and significance levels are given

^#^ *p* > 0.05; *p < 0.05; **p < 0.01; ***p < 0.001

## Discussion

It appeared that the biomass production of *C. reinhardtii* was significantly decreased (about 40 %) when grown in undiluted flue gas at about 17 % CO_2_ as compared with pure CO_2_ gas at a concentration of about 5 % or flue gas with a CO_2_ concentration of 6 %. In the present study, flue gas with a concentration of 10 % CO_2_ decreased the biomass by about 20 %. This is quite similar to the 20–25 % biomass reduction in the same strain recently found with flue gas from waste combustion at a concentration of 11.4 % CO_2_ (Mortensen and Gislerød 2014). Also, Bark (2012) found that 20 % CO_2_ was too high to give optimal growth of this species.

Similar results have been obtained with *Scenedesmus dimorphus*, where 20 % CO_2_ significantly reduced biomass production as compared to 2 and 10 % CO_2_ (Jiang et al. 2013). *Chlorella pyrenoidosa* could tolerate concentration up to 50 % CO_2_; however, the growth was the best at 10 % (Tang et al. 2011). Several microalgae species have shown good tolerance to concentrations up to approximately 20 % and even up to 100 % (van den Hende et al. 2012). In the present experiment, CO_2_ stress that inhibited the efficiency of photosystem II might explain the growth reduction at high CO_2_. Although the pH of the culture was slightly lower at the highest flue gas concentration, this did not seem to be the reason for the reduced growth, since the pH still was within an optimal range of this species (Bark 2012). Olaizola (2003) concluded that a moderate decrease in pH caused by flue gases probably has a small effect on the growth of microalgae cultures. Nevertheless, the use of a bicarbonate buffer in the present study avoided a strong acidification that would otherwise have arisen at high CO_2_ concentrations. In the present experiment with flue gas, the NO_*x*_ level reached about 100 ppm and the SO_2_ level 1 ppm in combination with 17 % CO_2_. Such concentrations, however, seem seldom to affect the growth of microalgae (Brown 1996; Olaizola 2003; Hende et al. 2012; Farrelly et al. 2013; Jiang et al. 2013).

Kliphuis et al. (2011) found a 20 % increase in growth rate in *C. reinhardtii* when air with 21 % O_2_ was replaced by N_2_ (0 % O_2_) when mixed with 2 % CO_2_. This effect was attributed to the reduction in the oxygenase activity of Rubisco, resulting into decreased photorespiration and increased growth. However, Vance and Spalding (2005) who studied the same species over a range of CO_2_ concentrations (0.005–5 %) at 2 and 20 % O_2_ found no discernable effect of the O_2_ concentration on growth. The effect on growth was related to the CO_2_ concentration alone. Unfortunately, the results with the present flue gas were in accordance with the conclusions of Vance and Spalding (2005) and not Kliphuis et al. (2011). The very low O_2_ concentration (down to about 1 %) in the flue gas from the silicomanganese smelter resulting into dissolved O_2_ values in the algae culture down to about 4 mg L^−1^ seemed therefore not to be of any particular benefit. In addition, the very high CO_2_ concentration (up to 20 %) resulting into dissolved CO_2_ values of up to 490 mg L^−1^ in algal culture caused a negative effect on growth. Based on the results from the present experiments, it can be concluded that flue gas containing up to about 6 % CO_2_ would be optimal for the production of the present *C. reinhardtii* strain. However, the special properties of the flue gas from the silicomanganese smelters seemed not to provide an advantage over other flue gas sources.
